# DNA Uptake by Type IV Filaments

**DOI:** 10.3389/fmolb.2019.00001

**Published:** 2019-02-05

**Authors:** Kurt H. Piepenbrink

**Affiliations:** ^1^Department of Biochemistry, University of Nebraska-Lincoln, Lincoln, NE, United States; ^2^Department of Food Science and Technology, University of Nebraska-Lincoln, Lincoln, NE, United States; ^3^Nebraska Food for Health Center, University of Nebraska-Lincoln, Lincoln, NE, United States; ^4^Center for Integrated Biomolecular Communication, University of Nebraska-Lincoln, Lincoln, NE, United States

**Keywords:** natural competence, type IV pili, horizontal gene transfer, competence pili, Flp pili, DNA-binding

## Abstract

Bacterial uptake of DNA through type IV filaments is an essential component of natural competence in numerous gram-positive and gram-negative species. Recent advances in the field have broadened our understanding of the structures used to take up extracellular DNA. Here, we review seminal experiments in the literature describing DNA binding by type IV pili, competence pili and the flp pili of *Micrococcus luteus*; collectively referred to here as type IV filaments. We compare the current state of the field on mechanisms of DNA uptake for these three appendage systems and describe the current mechanistic understanding of both DNA-binding and DNA-uptake by these versatile molecular machines.

## Introduction

The explosion of genomic data available from high-throughput sequencing and the rising burden of antimicrobial resistance in pathogenic bacterial species have generated a great deal of interest in horizontal gene transfer, the process by which genetic material is transmitted from one species to another. Horizontal gene transfer is thought to occur primarily through the transmission of mobile elements in plasmid conjugation systems and bacteriophages. But many bacteria produce molecular machinery to specifically transport DNA from extracellular space into the cytoplasm where it can be incorporated into their own chromosomal DNA. The ability of bacteria to take up extracellular DNA and to make use of the genetic information contained within it (competence) is well-documented in the annals of microbiology. Famously, the natural competence of *Streptococcus pneumoniae* was instrumental in identifying DNA as the genetic material by Oswald Avery, Fredrick Griffith and others.

With one notable exception (Hofreuter et al., 2001), the protein assemblies responsible for DNA-uptake are type IV pili or structurally-related protein fibers (Bovre and Froholm, 1971; Biswas et al., 1977; Breitling and Dubnau, 1990; Porstendörfer et al., 1997, 2000; Chung and Dubnau, 1998; Graupner and Wackernagel, 2001; Graupner et al., 2001; Aas et al., 2002b; Friedrich et al., 2002, 2003; Meier et al., 2002; Averhoff and Friedrich, 2003; Meibom et al., 2005; Chen et al., 2006; Laurenceau et al., 2013; Angelov et al., 2015; Antonova and Hammer, 2015; Karuppiah et al., 2016). Type IV pili are fibers composed of protein subunits extracted from the inner/plasma membrane and secreted from the cell and noncovalently assembled into a helical fiber in a conserved pattern. They universally assemble with N-terminal transmembrane helices packed together in the center of the fiber with soluble C-terminal domains on the surface. Com and Flp pilins have homologous N-terminal domains but divergent C-termini and are thought to form similar fibrous structures. In this review, we refer to these extracellular appendages collectively as type IV filaments (T4F). Type IV filaments, including type IV pili, the competence pili (or pseudopili) of gram-positive bacteria (notably *Staphylococcus, Streptococcus*, and *Bacillus* species) and the recently-discovered Flp/tad-like pilus of *Micrococcus luteus* are united by their use of several homologous proteins; (i) pilin proteins which contain an N-terminal transmembrane-like helix and can be extracted from the inner/plasma membrane and assembled into extracellular helical fibers, (ii) AAA+ ATPase proteins (or **A**TPases **A**ssociated with diverse cellular **A**ctivities) (Frickey and Lupas, 2004; Iyer et al., 2004) responsible for extension and/or retraction of the filament by the transfer of pilin subunits from the inner/plasma membrane to the pilus (or *vice versa*) and (iii) an integral membrane protein of the inner/plasma membrane which is essential for pilus biogenesis.

The mechanisms by which these protein assemblies mediate DNA-transport have been explored by many research groups over decades but remain somewhat mysterious. Here we introduce the prevailing models of uptake in well-studied gram-negative and gram-positive bacterial model systems with an emphasis on recent developments in structural biology which may shed light on the (potentially diverse) molecular mechanisms of natural competence.

## Macromolecular Assemblies of Type IV Filaments

Similar type IV filaments are produced by both gram-negative and gram-positive bacterial species despite the differences in peptidoglycan and membrane structure. Type IV pili and tad-like pili are found in a variety of both gram-negative and gram-positive species while competence pili have, thus far, only been found in gram-positive bacteria. However it is worth noting that until fairly recently, type IV pili were almost unknown in gram-positive bacteria but are now known to be widely-distributed in some genera (Melville and Craig, 2013; Piepenbrink and Sundberg, 2016).

**Figure 1:**
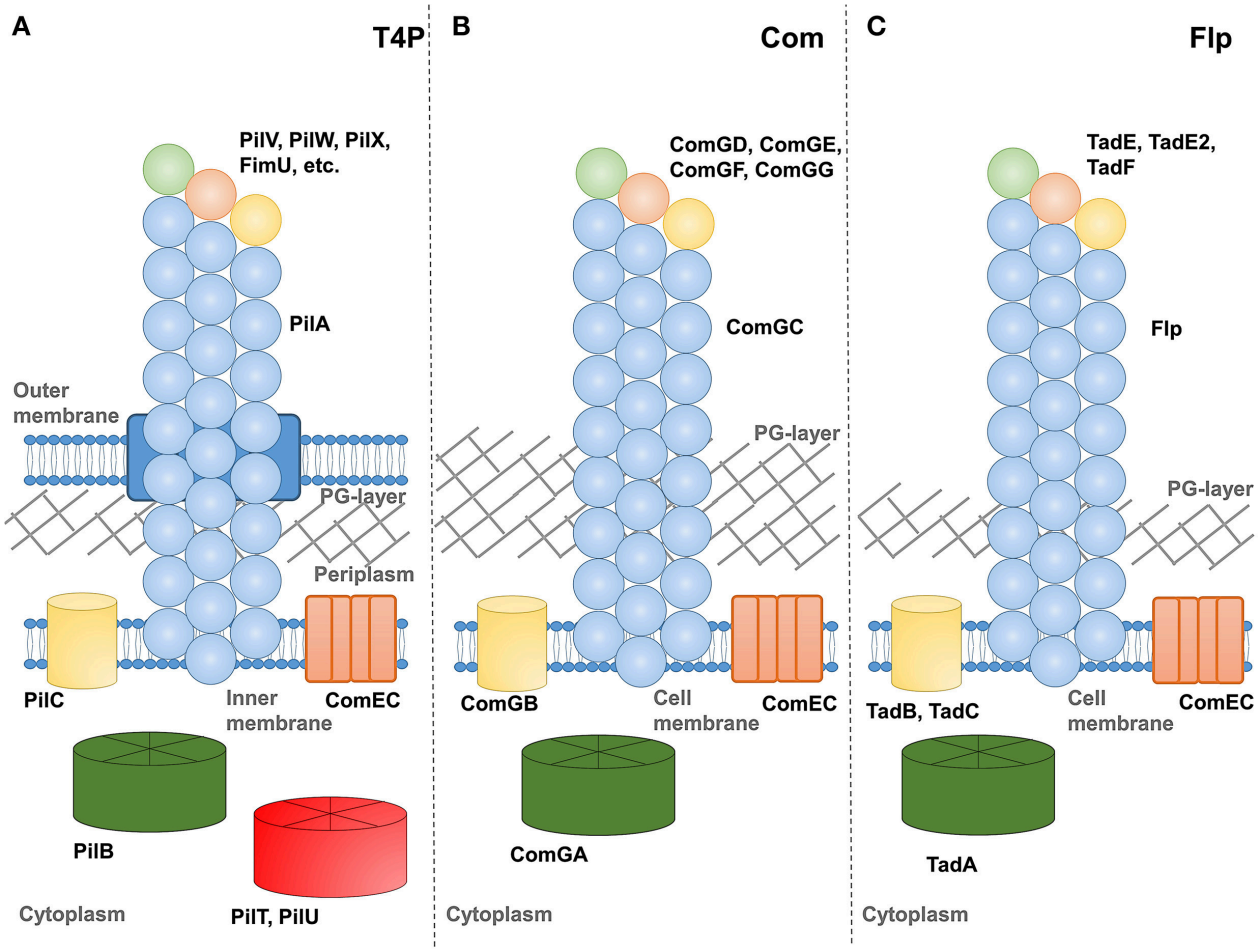
Schematics of T4F systems. Major pilin proteins are depicted in blue, minor pilin proteins in green, orange and yellow, putative extension ATPase proteins in green, retraction ATPases in red, PilC homologs in yellow and ComEC in orange. **(A)** type IV pili from a Gram-negative species, **(B)** competence pili from a Gram-positive species and **(C)** Flp pili from *M. luteus*.

Figure 1 shows schematic representations of (a) type IV pilus (T4P), (b) competence, and (c) tad-like filament systems. Each includes at least one assembly ATPase in the cytoplasm, an integral membrane protein and several pilin-like proteins. One pilin, referred to as the major pilin in type IV pili, is thought to predominate and form the vast majority of the pilus fiber. While all T4F systems include at least one other pilin protein (identified by the combination of an N-terminal proteolysis site, transmembrane domain and soluble C-terminal region), their functions are less well-understood. Speculatively, we depict them here forming an initiation complex at the pilus tip in a manner similar to models proposed for *Pseudomonas aeruginosa* type IV pili (Nguyen et al., 2015), the toxin-coreggulated pilus TCP) of *Vibrio cholerae* (Ng et al., 2016) and the type II secretion system of enterotoxigenic *Escherichia coli* (Korotkov and Hol, 2008). In at least one case, multi-domain minor pilin subunits can also be found incorporated sporadically along the pilus length of a type IV pilus (Piepenbrink et al., 2015).

Another notable gap in our understanding relates to the structure of the pore by which extracellular appendages transverse the peptidoglycan (PG) layer of gram-positive bacteria. In Figure 1, we depict a channel extending from a periplasmic-like space at the surface of the plasma membrane through the PG layer into extracellular space, analogous to the secretin protein used by numerous appendages in gram-negative bacteria (D'Imprima et al., 2017). Previous authors have varied in their depictions from a channel-like structure (Melville and Craig, 2013) to a simple gap in the PG layer (Muschiol et al., 2015).

Our understanding of the process by which these pili are extended and (in at least in some cases) retracted remains incomplete but involves some signal from the cytoplasmic ATPase proteins being transmitted across the membrane where pilin proteins, resident by virtue of their N-terminal transmembrane helices, can be extracted from the membrane and incorporated into a pilus. Because the pilin proteins and cytoplasmic ATPase proteins are similar enough in all three (T4P, com, tad/flp) systems that we can identify them as homologs by sequence alignment, we might presume that the mechanism of transmission across the membrane is also similar. However homologs for many proteins known to be essential for the assembly of type IV pili (PilM, PilN, PilO, PilP) are absent or unidentifiable in com pilus operons. The integral membrane protein, PilC or ComGB, does appear to be universal in T4F systems.

## Involvement of Type IV Filaments in Natural Competence

### Type IV Pili

Of the three filamentous systems discussed here, type IV pili (T4P) are by far the best-studied. Although they are found in both Gram-positive (Melville and Craig, 2013; Piepenbrink and Sundberg, 2016) and Gram-negative species (Craig and Li, 2008; Giltner et al., 2012) where they have been found to mediate host-cell adhesion, biofilm formation and twitching motility (Chiang and Burrows, 2003; Piepenbrink et al., 2016; Das et al., 2017), they have only been found to mediate natural competence in Gram-negative species. Substantial bodies of literature exist studying the role of T4P in natural competence in *Vibrio* species (Bartlett and Azam, 2005; Seitz and Blokesch, 2013; Antonova and Hammer, 2015), *Acinetobacter* (Herzberg et al., 2000; Porstendörfer et al., 2000; Harding et al., 2013), *Thermus thermophilus, Haemophilus* and *Neisseria* and this relationship has also been demonstrated in *Legionella* (Stone and Kwaik, 1999) and *Moraxella* (Luke et al., 2004). The multiplicity of functions for T4P suggests that conflicting evolutionary pressures may lead to specialization even within a single species. Recently we reported functional differentiation within the T4P of *Acinetobacter baumannii* REF apparently driven by a trade-off between twitching motility and biofilm formation. We propose that these differences are driven by differences in the formation of bundles of T4P from neighboring bacterial cells. We are currently investigating the effects of these differences on natural competence, which, like twitching motility, requires pilus retraction but also requires DNA-binding, which has been proposed to nucleate biofilm formation (Ronish et al., 2019).

Type IV pili are commonly divided into two subtypes; type IVa are distributed throughout Gram-negative bacterial genera and are widely studied in *Pseudomonas, Neisseria* and *Vibrio cholerae* Mannose-sensitive hemagglutinin (MSH) pili. Type IVb are primarily found in enteric bacteria, including Salmonella, several pathogenic E. coli species and *Vibrio cholerae* Toxin-corregulated pili (TCP) (Craig and Li, 2008). When compared to their type IVa counterparts, they have larger major pilin subunits, leading to wider fibers and longer N-terminal signal sequences). Some previous classification systems placed tight adherence (tad) pili in type IVb despite the small size of their major pilin proteins (Giltner et al., 2012), but recently Ellison et al., proposed granting them their a separate category, type IVc pili (Ellison et al., 2017). For the purposes of this review, we treat them as a separate entity below. Notably, while there are many cases of type IVa systems inducing natural competence; to date no type IVb pili have been shown to do so.

The involvement of type IV pili in natural competence has been demonstrated by mutagenesis studies showing that deletions of the major pilin (typically *pilA* or *pilE*), the extension ATPase (*pilB*) or the retraction ATPase (*pilT*) are deleterious for natural competence. Notably the major pilin (PilA-homolog) in *Acinetobacter bayli* is referred to as ComP and should not be confused with the ComP of *Neisseria* (see below). However the connection between the pili themselves and natural competence is less clear; in many cases competence exists under conditions in which few or no type IV pili are visible by transmission electron microscopy (Rudel et al., 1995; Long et al., 2003). For *Neisseria*, this has led to speculation that an alternate structure exists in which uses components of the type IV pilus system but extends little, if any, beyond the outer membrane. Notably, recent results demonstrate that in *Neisseria*, truncations of the major pilin, PilE, up to a cleavage site for release of the soluble C-terminal portion (S-pilin) allow for natural competence but not visible pili and mutations of the proteolysis site abolish natural competence, leading to the suggestion that some competence structure is formed from the N-terminal fragment of PilE (PilE-Ntd) (Obergfell and Seifert, 2016).

Unlike competence pili, type IV pilus systems typically include multiple cytoplasmic ATPase proteins; not only an extension ATPase (PilB) but also 1-2 others thought to mediate pilus retraction (PilT, PilU, or PilT2). Mutations to or deletions of *pilT* have been found to negatively impact transformation in several species (Wolfgang et al., 1998a,b; Aas et al., 2002b; Meier et al., 2002; Harding et al., 2013). In *Pseudomonas stutzeri*, deletion of *pilU* resulted in a 90% reduction in natural transformation (Graupner et al., 2001) while the equivalent mutation had no effect on transformation in *Vibrio cholerae* (Seitz and Blokesch, 2013).

**Figure 2:**
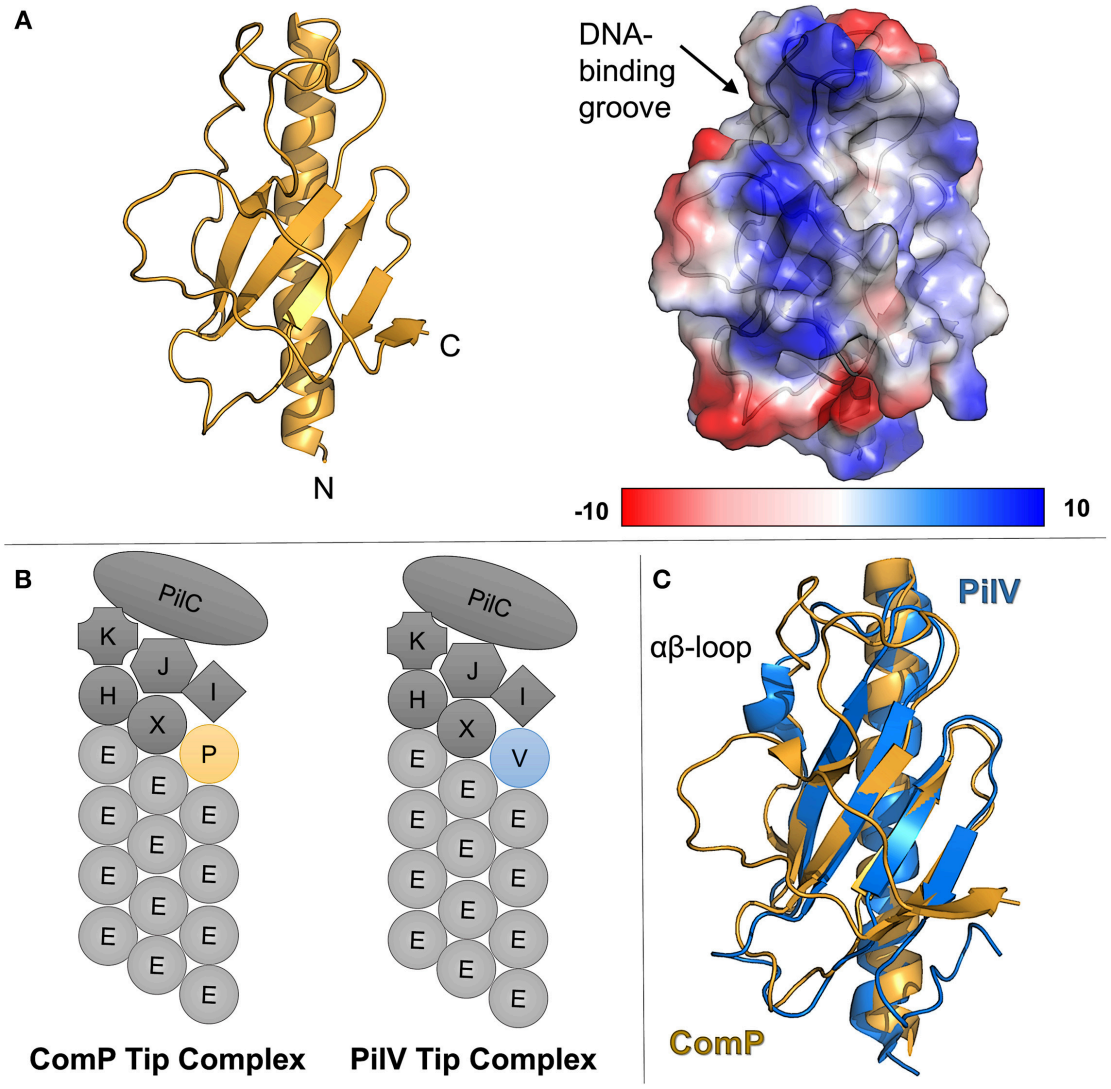
ComP, the pilin DNA receptor from *Neisseria*. **(A)** (left) ribbon diagram of ComP in gold, (right) columbic electrostatic surface calculation of ComP, **(B)** schematics of *Neisseria* type IV pili showing the major pilin, PilE, as well as a putative tip complex containing PilK, PilJ, PilI, PilH, PilX, PilC, and either ComP or PilV. **(C)** Superimposition of ComP (gold) and PilV (blue).

Uniquely, in the *Neisseria* type IV pilus system, the extracellular DNA receptor has been identified as a minor pilin, ComP (Koomey, 1998; Wolfgang et al., 1999; Cehovin et al., 2013). Figure 2A shows the structure of ComP from *Neisseria meningitidis* (Berry et al., 2016). ComP has a higher affinity for sequences containing DUS (DNA uptake sequence), a conserved short DNA sequence (GCCGTCTGAA in *Neisseria*) preferentially taken up during natural competence (Chen and Dubnau, 2004; Findlay and Redfield, 2009). This preference has been used to explain the relative abundance of DUS-containing sequences in horizontal gene transfer (Cehovin et al., 2013). Despite their similarity in other respects, homologs of ComP have not been identified in other commonly-studied type IVa pilus systems, suggesting that other DNA-binding minor pilins significantly divergent or that other DNA receptors are utilized.

The deletion of *comP* abrogates natural competence in Neisseria but does not affect the level of pilation (Wolfgang et al., 1999). Similarly, the deletion of another minor pilin gene, *pilV*, leads to a phenotype which is pilated, but shows reduced host-cell adhesion (Winther-Larsen et al., 2001). However the deletion of both *comP* and *pilV* leads to a non-pilated mutant (Aas et al., 2002a). This supports a model in which a tip complex in Neisseria type IV pili can include either PilV or ComP but not both (Figure 2B).

A comparison of the ComP and PilV crystal structures (Figure 2C), shows differences in structure, particularly in regions expected to be exposed in the pilus fiber, but substantial similarity in the overall fold. Figure 2C shows the superimposed structures of ComP (gold, PDBID: 5HZ7) and PilV (blue, PDBID: 5V0M). Both proteins show the proto-typical type IV pilin fold, a central α-helix (α1-C) followed by a loop leading into an antiparallel β-sheet, but ComP has no α-character beyond that initial helix while PilV has a short α-helix in the αβ-loop. The C-terminal regions also differ as ComP has an additional strand in the central β-sheet. The similarity in the loop length and conformation for the loops between the 1st and 2nd, 2nd and 3rd and 3rd and 4th β-strands is noticeable. But because the αβ-loop and C-terminus are typically the regions which mediate pilin-headgroup interactions in models of pilus fibers, we expect that structural rearrangements would be required for pilin-headgroup proteins capable of binding either Comp or PilV.

### Competence Pili

Analogous to type IV pilus systems, the competence pili (or pseudopili) are composed primarily of a single protein polymerized into a helical fiber. This major pilin (ComGC) can be isolated from the supernatant or membrane-bound fractions of *Bacillus subtilis*. The minor pilins, ComGD, ComGE, ComGF, and (in some species) ComGG, are required for competence in *S. pneumoniae* but ComGG was not detected in sheared pilus fractions by Balaban et al. (Muschiol et al., 2015).

**Figure 3:**
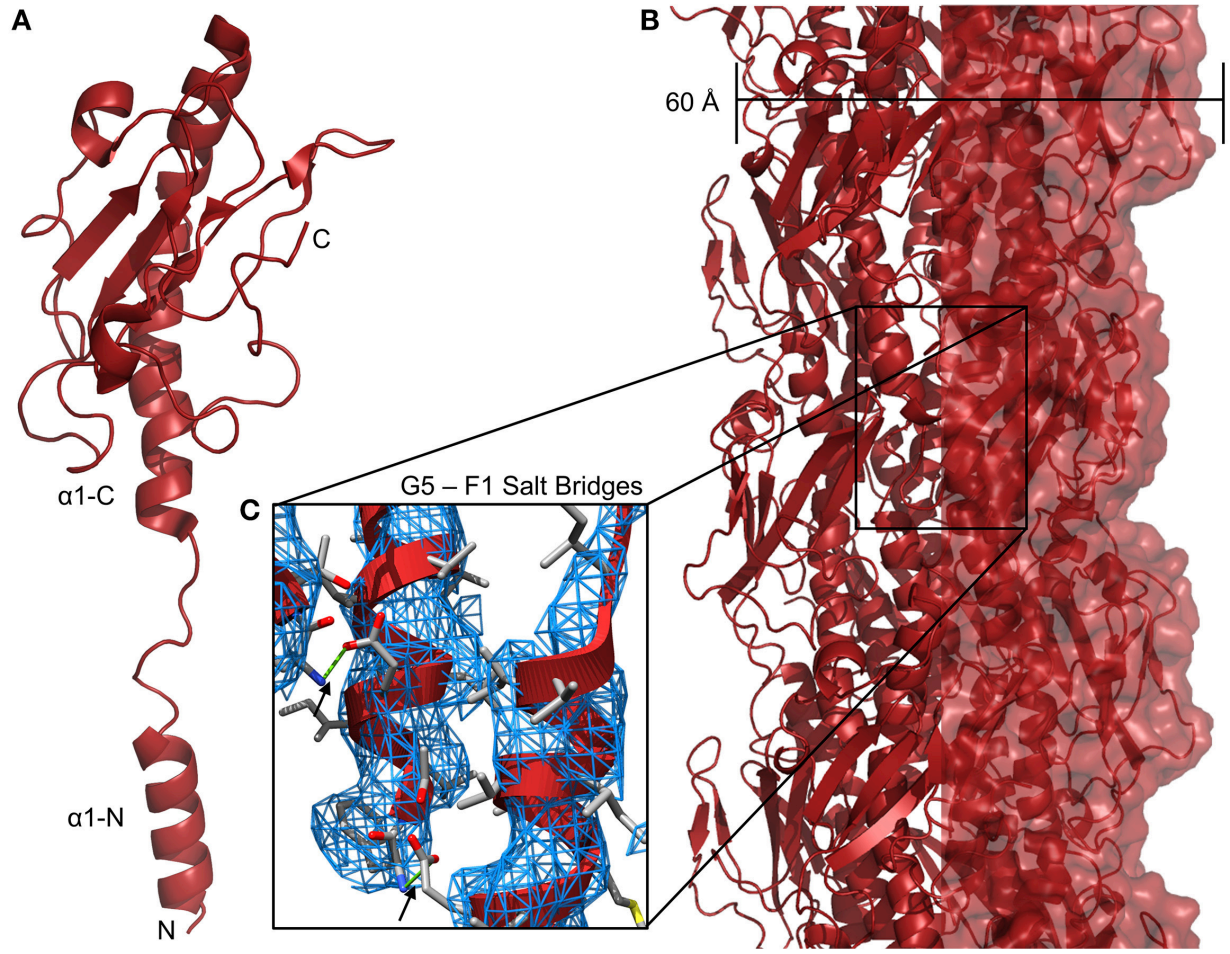
*Neisseria gonnorheae* type IV pili. **(A)** Ribbon diagram of PilE monomer. **(B)** Structure of a *N. gonnorheae* type IV pilus (PDBID: 5VXX). **(C)** Salt bridges between the side-chain of glutamate 5 and the amino terminus (F1) of the i + 1 pilin, 3DEM electron density is shown as a blue mesh.

Although no sequence similarity exists between the T4P and com pilins beyond the N-terminal α1-N helix, two factors suggest that they form similar filamentous structures. One is the similarity of the AAA+ ATPase proteins TadA, ComGA, and PilB; similarities in the extension machinery imply similarity in substrate. The other is the conservation of a glutamate residue at position 5 of the mature protein (e.g., with the signal peptide removed). In models of type IV pili, this glutamate forms a salt-bridge with the N-terminus of the previously-extended pilin subunit (i.e., the next protein in the fiber going from the membrane to the tip) (Craig et al., 2004) (Figure 3C). In the *Bacillus subtlis, Streptococcus pneumoniae*, and *Staphylococcus aureus* com systems, all putative pilins save one contain a glutamate at position 5 and similar patterns are observed in type IV pili and type II secretion (T2S) systems. The ternary structure of three minor pilins from the *E. coli* type II secretion complex, GspI, GspJ, and GspK, shows the three assembled into a helix similar to the helical fiber of type IV pili with GspK at the apex (Korotkov and Hol, 2008). Because GspK does not have a glutamate at position 5 and is at the apex of the pilus where there is no “i + 1” pilin to form a salt bridge with, one might expect that no T4F or T2S structures would include more than pilin without a glutamate at position 5 and that any pilin without glutamate 5 would be a minor pilin only incorporated during pilus initiation at the tip. Table 1 shows all putative pilin proteins from selected *A. baumannii* (T4P), *S. pneumoniae* (com), and *M. luteus* (tad) strains; in each no more than one has a non-glutamate residue at position 5. We hypothesize that these two proteins, PilX in *A. baumannii* and ComGF in *S. pneumoniae* would be found at the tips of their respective pili.

**Table 1:** Pilin N-terminal sequences.

**Protein**	**Amino acid sequence**
***Acinetobacter baumannii AYE***	
PilA	MNAQKGFTLIELMIVVAIIGILAAIAIP…
FimU	MRGIIPQEGFTLVELMVTIAVMAIIALMAAP…
PilV	MHHYKQKAQAGVGLLEVLVALILLAIGVLGYV…
PilW	MAQSFISNKEQGFTLIELIVALALGLILVAAATQLF…
PilX	MTHLNYKNIKGRSKETGATLIVVLIILLIVISVGVLAIRV…
PilE1	MNKYYILRFNQGFTLIELMVVIVIVAIFASIAIP…
PilE2	MKNGFSLIEIMVVVAIVAILAAIATP…
***Streptococcus Pneumoniae*** **R6**	
ComGC	MKKMMTFLKKAKVKAFTLVEMLVVLLIISVLFLLFVP…
ComGD	…MIKMEEQIVKSMIKAFTMLESLLVLGLVSILALGLSG…
ComGE	…VQNSCWQSKSHKVKAFTLLESLLALIVISGGLLLFQA…
ComGF	MWKKKKVKAGVLLYAVTIAAIFSLLLQFYLNR…
***Micrococcus luteus trpE16***	
Flp	…AAGEETGAITAEYGILTLAAVGFAGVLAVVLTSP…
TadE	MGVRWRDDRGAAVAESTMVMTLVVLLFAALLQAGVV…
TadF	MRSVNDVRDERGAATVEFLGLTLVLLIPVVYLMIYV…
TadE2	…GWSRGGETGAVTAEYAVLLPVIAFVLVSVLLAGAAAV…

*N-terminal amino acid sequences for putative pilin proteins from T4P, com and Flp pilus systems. N-terminal signal peptides (removed to create the functional protein) are depicted in blue. The glutamate residues typically found at position 5 are depicted in red*.

No DNA receptor has been identified for com pili but Laurenceau et al., demonstrated that the *S. pneumoniae* type IV filament binds DNA at many points along its length (not simply at the tip as has been suggested for *Neisseria*) (Laurenceau et al., 2013). However *S. pneumoniae* ComGC (the major pilin) does not bind DNA monovalently (Balaban et al., 2014). One possible explanation is that the DNA-binding surface is formed at the interface of the ComGC subunits and hence found in the assembled pilus but not the individual pilin. Alternatively, one of the minor pilin subunits (ComGD, ComGE, or ComGF) may be incorporated throughout the length of the pilus rather than at the tip as has previously been described for the minor pilin PilJ in *Clostridioides* (*Clostridium*) *difficile* type IV pili (Piepenbrink et al., 2015).

### Tad/Flp Pili

Recently, Angelov and colleagues described the natural competence system of *Micrococcus luteus*, a Gram-variable member of the *Actinobacteria*. The *M. luteus* genome contains homologs for *comEA* and *comEC* but not the *comGA-F* cluster. Instead, *M. luteus* produces pili related to the Flp (fimbrial low-molecular-weight protein) pili of *Aggregatibacter actinomycetemcomitans*, which are required for natural transformation (Angelov et al., 2015). Additionally, another member of *Actinobacteria, Cutibacterium* (formerly *Propionibacterium*) *acnes*, has also been found to carry a plasmid containing genes encoding a tad/flp pilus system; strains containing the plasmid were found to express pilus-like appendages by SEM, while a plasmid negative strain did not (Davidsson et al., 2017).

*A. actinomycetemcomitans* is a gram-negative periodontal pathogen in which the tad (tight adherence) locus was first identified. This locus is necessary for surface adherence and biofilm formation through the production of Flp pili which are commonly viewed as a subset of type IV pili (Ellison et al., 2017) but have also been proposed as an orthologous structure to the archaeal flagellar biosynthesis system (Tomich et al., 2007). These tad/flp systems can be found in a variety of Gram-positive and Gram-negative bacterial species, including *Pseudomonas aeruginosa, Burkholderia pseudomallei*, and *Caulobacter crescentus*. Flp pili form bundled fibrils commonly observed to be laterally associated with cells, which may offer some hint as to the mechanism by which they promote biofilm formation.

Two tad-like gene clusters can be found in *M. luteus*, each containing putative ATPase proteins (TadA, homologous to PilB) and putative minor pilins (TadE, TadF), one cluster also contains an *flp* gene, the product of which can be isolated from extracellular sheared pilus fractions. Based on immunofluroesence microscopy, Flp pili can be observed on the surface of *M. luteus*, but only on a minority of cells, even under competence-inducing conditions, and these pili appear to be shorter and less bundled than those observe in *A. actinomycetemcomitans* and *C. crescentus*.

## Type IV Filament Structures

Because of the similarity of the N-terminal portions of archeaeal flagellar subunits, type II secretion pseudopilins and type IV, com and Flp pilus subunits it is commonly hypothesized that all form similar helical fibers, varying considerably in length but similar in the pattern of assembly. However near-atomic resolution structural studies on intact fibers have been limited, thus far, to type IV pili (Wang et al., 2017) and archaella (Braun et al., 2016; Poweleit et al., 2016), and even studies of the individual subunits have produced many structures of type IV pilins, but only one structure from a com pilin and none at all of Flp pilins. To compare the structures formed by these types of pili, we have created models of the pili all of the proposed T4F fibers. Figure 3 shows the high-resolution structure of *Neisseria gonnorheae* PilE from a cryo-electron micrograph (Figure 3A). The cryo-EM reconstruction of the N. gonnorheae type IV pilus (Figure 3B) is ~60 Å wide, similar to what has been seen for lower resolution TEM measurements of other type IVa pili (Gold et al., 2015) and stabilization can easily be understood through the hydrophobic effect as the hydrophobic α1-N helices are packed together in the center of the fiber with the hydrophilic surfaces of the C-terminal headgroup exposed on the surface. The structure shown here is in no way general for all *Neisseria* T4P; particularly because class II *Neisseria meningitidis* T4P are hyperglycosylated, containing as many as five glycosylation sites per monomer (Gault et al., 2015). The recent high-resolution structure from Wang et al., shows that the α1-N helix melts in at least some conditions during polymerization (Wang et al., 2017). This is in contrast to high-resolution crystal structures of pilin monomers which show the helix intact, as we presume it to be when the pilin is inserted into the inner/plasma membrane (Forest et al., 1999; Craig et al., 2003, 2006; Hartung et al., 2011). In the models described below, we have modeled their α1-N helices based on the partially-melted 2017 model, hypothesizing that the same transition occurs.

**Figure 4:**
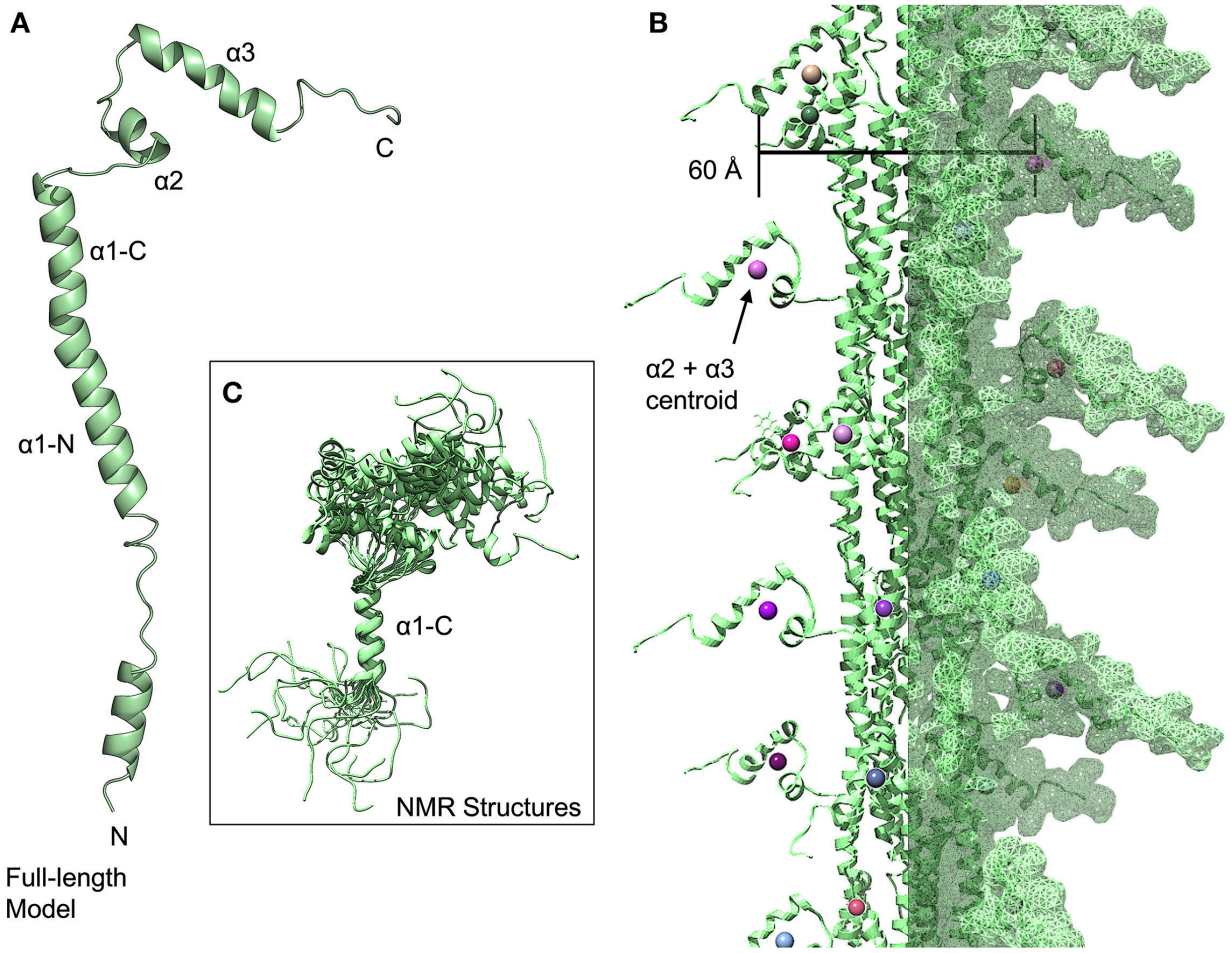
*Streptococcus pneumoniae* competence pili. **(A)** Ribbon diagram of a modeled full-length ComGC monomer (model 1). **(B)** Model of a *S. pneumoniae* pilus, centroids from the α2 and α3 helices are shown as spheres. **(C)** Superimposition of 20 models from the ComGC NMR structure (PDBID: 5NCA).

The NMR structure of the *S. pneumoniae* ComGC headgroup, the first structure of a pilin protein from a competence system (Muschiol et al., 2017), differs considerably from T4P despite the conservation of the hydrophobic α1-N helix because rather than the α1-C and β-sheet of the T4P headgroups, the headgroup consists of three helices (α1-C, which is a continuation of the α1-N, α2 and α3). In this model, we hypothesize that the latter portion of the α1-N helix and the α1-C helix form a continuous helix as they do in the two recent high-resolution electron micrographs of T4P (Wang et al., 2017) and in various crystal and NMR structures of individual type IV pilins (Craig et al., 2003; Hartung et al., 2011; Reardon and Mueller, 2013) (Figure 4A). However the first 39 amino acids of the mature PilE and ComGC proteins are only 24% identical; in time the structures of full-length com pilins may broaden these horizons. Notably, a large portion of the α1-C appears to be disordered in the NMR structure, which may indicate a greater dynamism than is seen for type IV pilins. The relative orientations of the three helices seen in the NMR structures is also highly dynamic (Figure 4C); a similar pattern was observed for the major pilin of *Geobacter sulfurreducens* (PDBID: 2M7G) (Reardon and Mueller, 2013). For our model of the assembled fiber, we have used model 1 from the NMR structure with presumption that there is in fact considerable variation in headgroup structure (Figure 4B). To represent this ensemble of possible headgroup structures, we can calculate a centroid of the atomic coordinates of the residues from these latter two helices; but of the 10 models in the NMR structure, some conformations would appear to be sterically disallowed in an assembled fiber. If we calculate a centroid of the α2 and α3 helices for each headgroup, based solely on model 1, and determine the width of the *com* pilus based on these centroids, we arrive at a width for the pilus of ~60 Å. This is consistent with the TEM measurement of 50–60 Å by Laurenceau et al. (2013).

**Figure 5:**
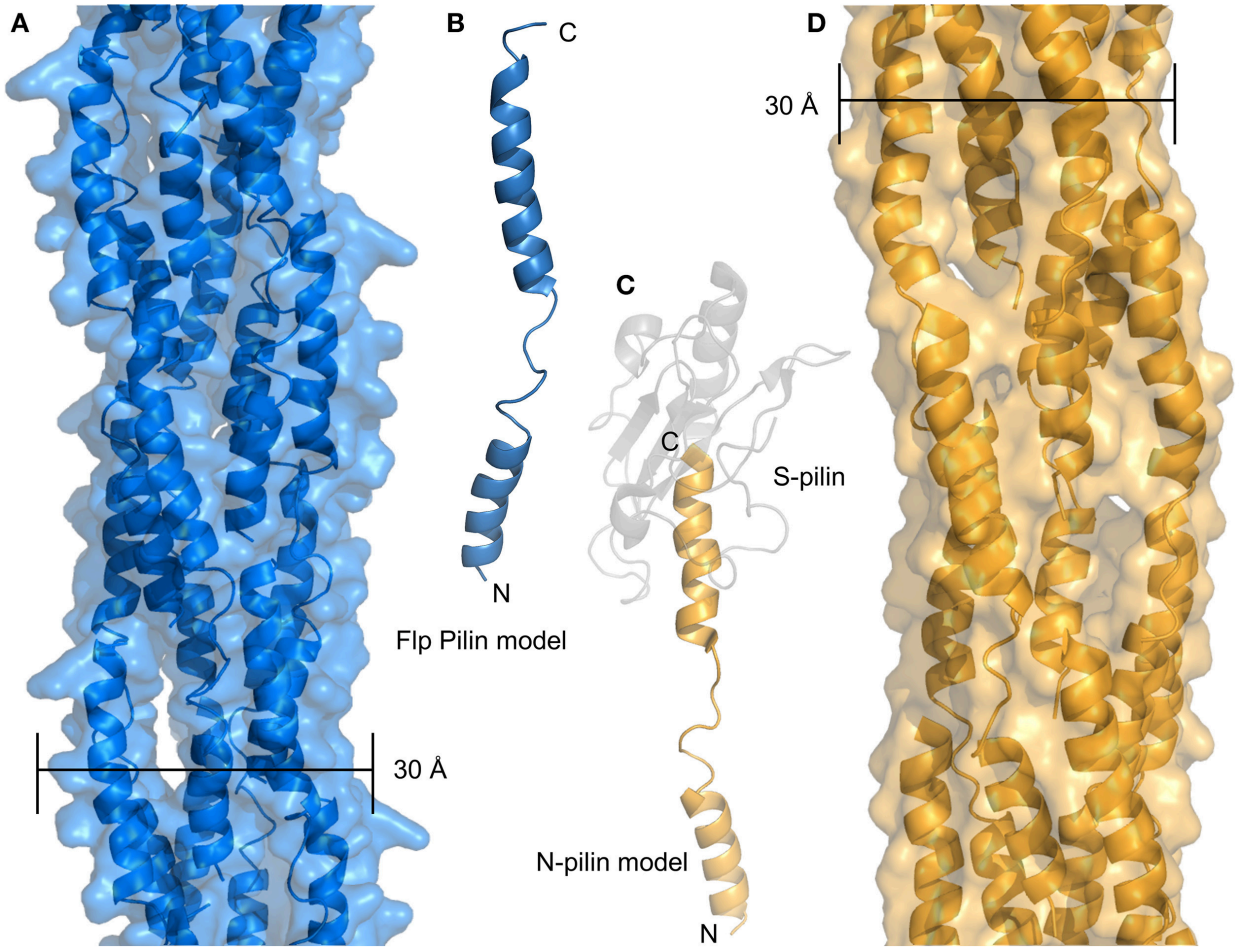
Minimal pilus filaments. **(A)** Model of an Flp pilus from *M. luteus*. **(B)** Model of a full-length monomer of the Flp pilin. **(C)** Model of the N-terminal portion of PilE after S-pilin cleavage. The N-pilin in depicted in gold, the S-pilin is shown in transparent gray. **(D)** Model of a filament composed of N-pilins.

Recently, evidence from *Micrococcus luteus* and *Neisseria gonorrhoeae* suggests that type IV filaments with minimal or no headgroups may mediate competence in some species. Figure 5A shows a model of the *M. luteus* Flp pilus based on the structure of the N-terminal portion of *N. gonnorheae* PilE (N-pilin or PilE-Ntd) (Figure 5B) The sequence identity between these sequences (20%) is lower than one would typically use for homology modeling, but the pattern of predicted secondary structure is consistent with our model and the study of type IV pili has frequently shown nearly identical structures from divergent sequences. Because the *M. luteus* Flp headgroup extends only a few residues past the α1-C helix, the resulting structure is predicted to be nearly identical to the N-pilin created upon cleavage of PilE at residue 39 of the mature protein (Figure 5C). The resulting pilus models for the Flp pilus and the hypothesized N-pilin fiber are both much more narrow than T4P, com or Flp pili which have been observed by cryo-EM, which are at least 50–60 Å for T4P and com pili (Giltner et al., 2012; Laurenceau et al., 2013) and anywhere from 40 to 80 Å for Flp pili (Skerker and Shapiro, 2000; Kachlany et al., 2001). This is despite the fact that all Flp pilins are much shorter (40–50 amino acids) than their T4P and com counterparts (which are 90+). Higher-resolution data on the structures of Flp pili will likely be necessary to resolve this apparent discrepancy but here we offer two possibilities: (i) another polypeptide may be associated with the α1-C of the Flp pilin subunits, essentially taking the place of the latter portion of the headgroup, or (ii) glycosylation with a large polysaccarhide could also increase the size of Flp subunits to something equivalent to T4P and com pilins. Notably, we predict that the N-pilin fiber proposed by Obergfell et al., would have a similarly thin diameter in the absence of some associating factor (Figure 5D) (Obergfell and Seifert, 2016).

## Extension and Retraction ATPases

**Figure 6:**
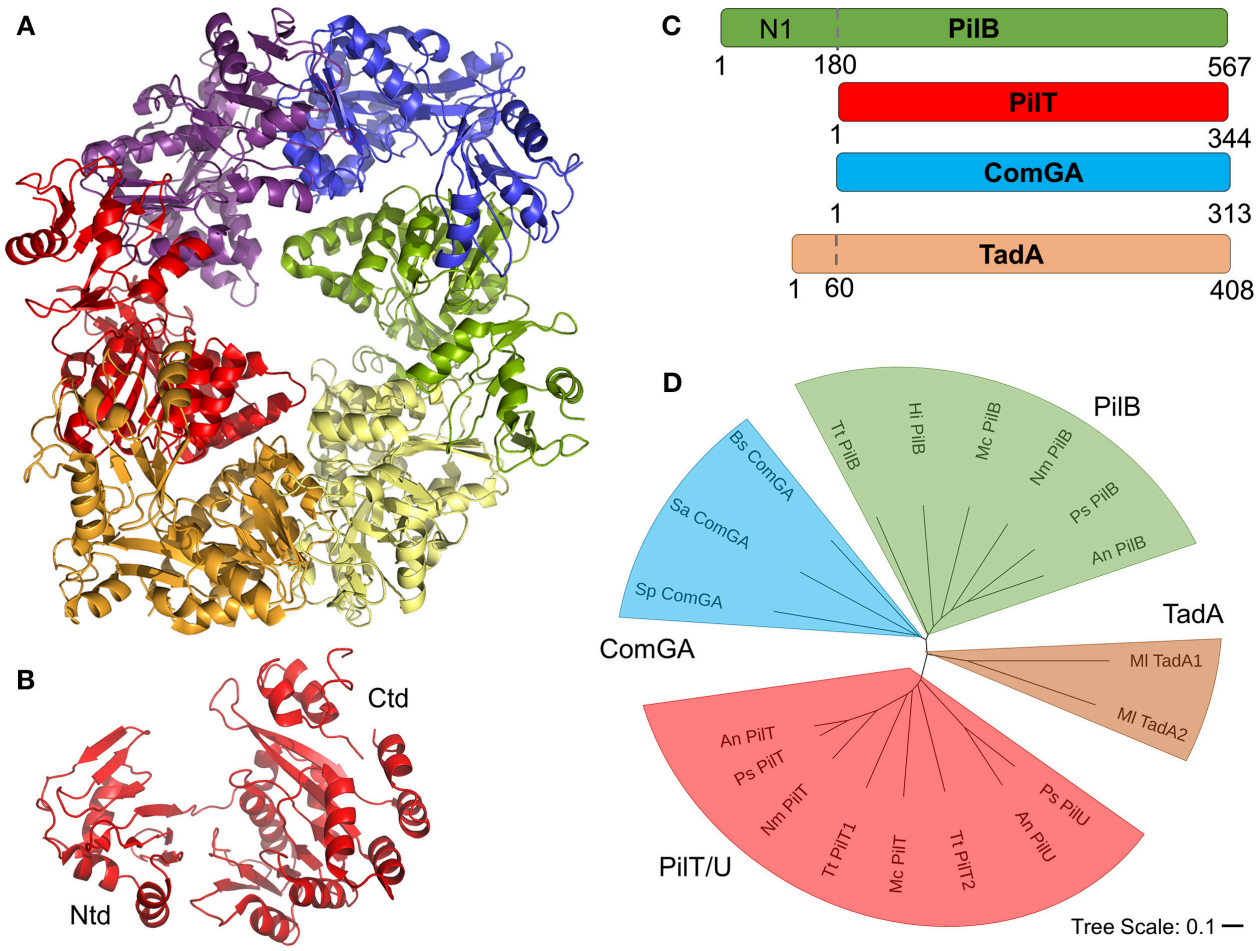
T4F ATPase motor proteins. **(A)** Structure of hexameric PilT from *Aquifex aeolicus* with each monomer in a different color (PDB ID: 2GSZ) **(B)** Monomer of PilT from panel A showing the two domains. **(C)** Aligned PilB (*P. stutzeri*), PilT (*P. stutzeri*), ComGA (*S. pneumoniae*), and TadA (*M. luteus*) sequences. **(D)** Phylogenetic tree of putative motor proteins showing PilB (from *T. thermophilus, H. influenza, M. catarrhalis, N. meningitidis, P. stutzeri*, and *A. nosocomialis*), ComGA (from *B. subtilis, S. aureus*, and *S. pneumoniae*), PilT/U (from *A. nosocomialis, P. stutzeri, T. thermophiles*, and *M. catarrhalis*), and TadA (*M. luteus*) branches.

Universally, the T4F systems described here contain at least one cytoplasmic AAA+ ATPase protein homologous to the PilB protein of T4P systems. Based on results from cryo-electron microscopy and tomography studies, primarily of PilB proteins, all of these motors are expected to form hexameric structures which can catalyze pilus extension and/or retraction through coupled ATP hydrolysis and rotation (Hospenthal et al., 2017). Recent studies of PilB and PilT have suggested that PilB and PilT may rotate in opposing directions, clockwise for PilB and counterclockwise for PilT, to mediate extension and retraction respectively (McCallum et al., 2017; Solanki et al., 2018). Figure 6A shows an x-ray crystal structure of PilT from Satyshur et al. (PDB ID: 2GSZ) (Satyshur et al., 2007), which crystallized in the hexameric complex expected to be formed by all homologous proteins. Figure 6B shows the monomeric protein consisting of two domains, a small N-terminal domain (Ntd) and a larger C-terminal domain (Ctd). PilB, PilT, PilU (T4P) ComGA (com) and TadA1 (Flp) are all similar enough in sequence to be easily identifiable even across very distantly-related bacteria; typically with sequence identities of ~30% across the length of the alignment (the Ntd and Ctd domains). However, these alignments show an N-terminal domain in PilB (commonly referred to as the N1 domain) which is entirely absent (PilT, PilU, ComGA) or severely truncated (TadA) in the other ATPase proteins (Figure 6C). Historically, ComGA and TadA proteins have been thought of as homologous of PilB rather than PilT, that is catalyzing extension but not retraction. However ComGA proteins, while similar in sequence to PilB over the two domains they hold in common, lack the PilB N1 domain; TadA, meanwhile contains what appears, based on the alignment, to be a truncated N1 domain, but its Ntd and Ctd do not clearly align with either PilB or PilT (Figure 6D).

The functions of ComGA and TadA proteins have implications for the mechanisms of DNA uptake because while retraction is a requirement for competence through T4P systems (Wolfgang et al., 1998a; Meier et al., 2002; Harding et al., 2013), there is currently no direct evidence for retraction of com or Flp filaments and no homologs of PilT/U proteins have been identified, despite their general conservation. This presents us with three possibilities, (i) retraction is required for DNA uptake by T4P, but not by com or Flp systems, (ii) retraction is required for DNA uptake by all T4F, but retraction can occur without catalysis from an AAA+ ATPase, as has been proposed for the Toxin-coregulated pilus (TCP) of *Vibrio cholerae* (Ng et al., 2016), or (iii) that ComGA and TadA proteins are capable of catalyzing both extension and retraction of their respective filaments. The latter hypothesis could explain the homology between PilB and PilT proteins as the ancestral gene from which *pilB, pilT, pilU, comGA*, and *TadA* and others evolved could have been an enzyme capable of catalyzing both extension and retraction (perhaps in complex with different cofactors) and this dual-activity could still be found in some AAA+ ATPase proteins.

## Models of DNA Uptake

Ultimately, the goal of investigations into pilus-biogenesis, the composition of pilus fibers and DNA-binding by type IV filaments centers around understanding the mechanism by which DNA is captured and brought into the cell, past the outer membrane (for Gram-negative species) and peptidoglycan layer. Conceptually, we can divide this mechanistic conundrum into two questions, (i) how does the pilus adhere to DNA? and (ii) how does DNA make its way past the cell's initial barriers and come into contact with ComEC? This mechanism may vary between species, meaning that the possibilities here are not intended to be entirely exclusive, but simply to describe the hypotheses which have been put forward thus far, extending them to include any logical alternatives.

**Figure 7:**
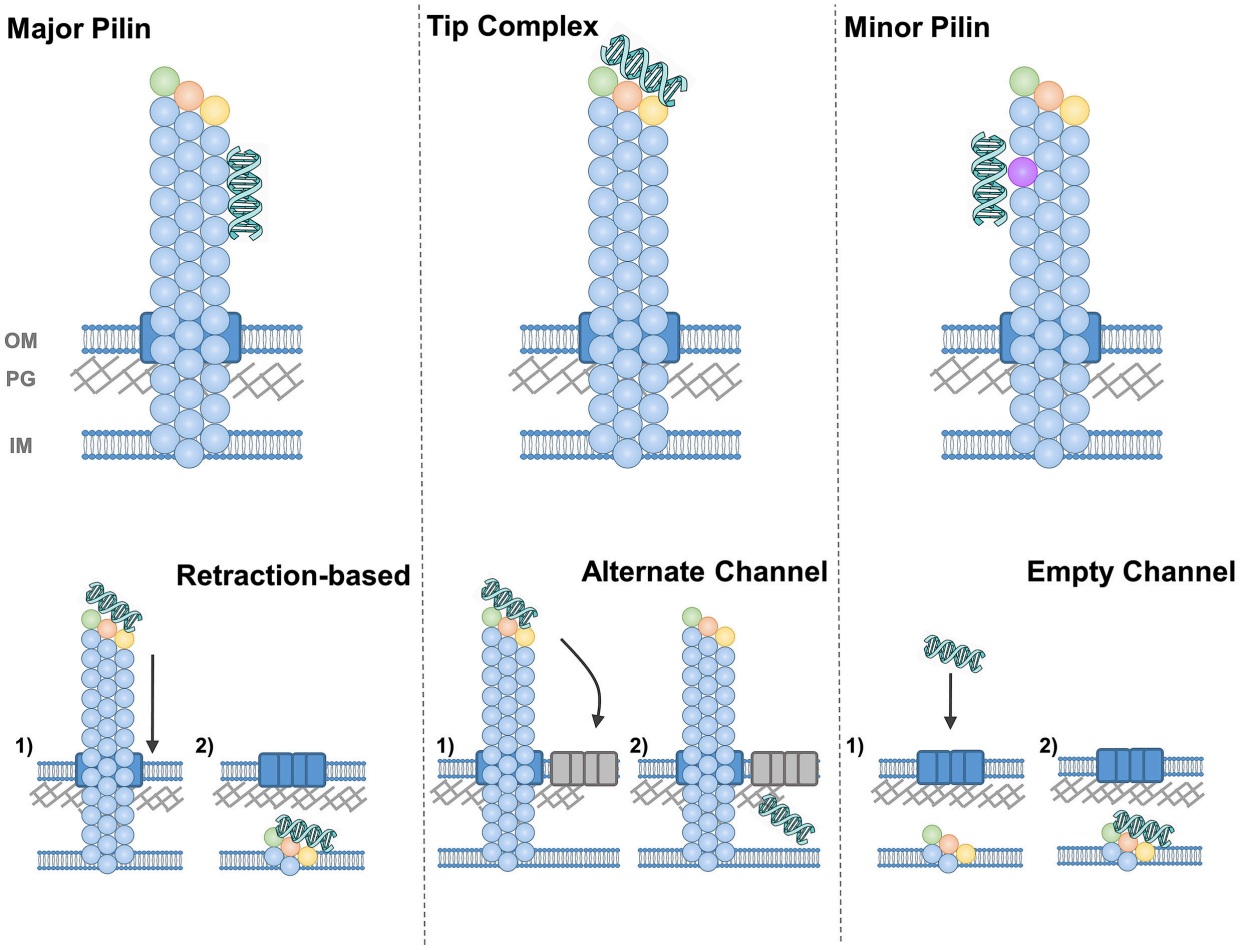
Diagrams of DNA-binding **(top)** and DNA-uptake **(bottom)** by T4F. DNA-binding is depicted by the major pilin (left), a tip complex (center) and minor pilins along the pilus length (right). DNA uptake is depicted by retraction through the secretin (or equivalent pore) (left), by diffusion through another channel (center) and by diffusion through an empty secretin-like channel (right).

The simplest answer to our first question is depicted in Figure 7 in the top left panel, that is, that the major pilin protein binds to DNA. This hypothesis has the advantage conceptually that the major pilin has been found to make up ~99% of the pilus fiber and hence takes up the vast majority of the potential interaction surface. The chief obstacle for the major pilin being the DNA receptor is that numerous studies using recombinant proteins have failed to find DNA binding but major pilin monomers. However the properties of protein oligomers may well be different than monomers; that is the DNA-binding surface may be formed by oligomerization or binding which is below the limit of detection in assays with monomeric protein may still be physiologically-relevant for fibers containing thousands of subunits.

An alternative hypothesis is that the DNA receptor is contained in a complex at the pilus tip (Figure 7). This model has been validated by direct evidence showing DNA-binding at the tips of *V. cholerae* (Ellison et al., 2018) and at *Pseudomonas aeruginosa* (which has a very similar T4P system to the competent *P. stutzeri*) (van Schaik et al., 2005). The Neisseria DNA receptor, ComP, is also commonly thought to be at the pilus tip, both because it is expressed at relatively low levels and because ComP and PilV appear to compete for a position within the pilus (Aas et al., 2002a). Binding at the tip may also be more compatible with the notion that pili retract, pulling DNA through the same channel that pili are extended through (see more below).

As a third possibility, one could imagine that minor pilin subunits incorporated sporadically throughout the pilus rather than only at the tip, as has been demonstrated for *C. difficile* and *P. aeruginosa* T4P (Giltner et al., 2010; Piepenbrink et al., 2015), could bind to DNA specifically. The top right panel of Figure 7 depicts this possibility with this minor pilin subunit depicted in violet. Like binding to the major pilin, this hypothesis can explain the binding of the DNA along the shaft of the *S. pneumoniae* competence pilus (Laurenceau et al., 2013), but has an added advantage in that monomeric ComGC does not show DNA binding (Balaban et al., 2014), suggesting the possibility of another DNA receptor.

For the second question, how the DNA makes its way from the pilus to the internal membrane of the cell, several possibilities have been proposed and there is particular difficulty for Gram-positive bacteria where it remains unknown how the pili are extended through the peptidoglycan (PG) layer. In lower panels of Figure 7, we depict all the possibilities for a Gram-negative species (e.g., with an outer membrane), with the secretin (PilQ) depicted in blue. We presume that some equivalent pore through the thicker peptidoglycan layer of Gram-positive bacteria must exist, distinct from the general cell surface, to allow the passage of a ~6 nm pilus or even ~2 nm dsDNA strands (Melville and Craig, 2013). In *S. pneumoniae*, two lytic enzymes involved in PG remodeling were found to be upregulated during competence (Peterson et al., 2004).

For type IV pili, where retraction is essential for competence, the prevailing hypothesis is that as pili are retracted into the cell, DNA is pulled along, passing through the outer membrane (and peptidoglycan layer) through the secretin as depicted in the lower left panel of Figure 7. This hypothesis has the advantage of simplicity and has been proposed for both com (Maier et al., 2004; Laurenceau et al., 2013) and T4P (Wolfgang et al., 1998a; Matthey and Blokesch, 2016) systems. But one implication is that if DNA binds along the length of the pilus, the pore through which retraction occurs must be able to accommodate the additional width of the DNA. In the case of secretin pores, the maximum width is difficult to determine; fusion of mCherry to the C-terminus of PilE abolished pilus formation in *N. meningitidis* (Imhaus and Duménil, 2014), implying that bulkier pilins could not be secreted through PilQ, but many T4P systems contain large putative pilin subunits [ex. TcpB and CofB are ~55 kd (Kolappan et al., 2015)] and the width of T4P fibers varies by more than 2 nm (Giltner et al., 2012).

One solution to the difficulty of envisioning a channel with a width variable by ~2 nm is to hypothesize that the DNA strand passes through the PG layer and/or outer membrane through a different channel than the pilus fiber. This hypothesis is more popular in Gram-positive bacteria (Johnston et al., 2014), perhaps because it does not require retraction; DNA binding alone, close to the cell surface, could promote diffusion through this alternate channel (depicted in the lower center panel of Figure 7).

Finally, as an alternative to the notion that the pilus pulls the DNA strand through its pore, Balaban et al. suggested that the pili shearing and shedding into the supernatant may create a space for the DNA strand to pass through (the “hole in the wall” model) (Balaban et al., 2014) (Figure 7, lower right panel). The production of an extracellular appendage solely as a means to create a gap in the PG layer seems on the face of it, to be a complex solution to a simple problem. However, T4P have been shown to mediate many distinct functions and the same could be true for com and Flp pili. If the evolution of a filament was driven by other factors, gaps in the PG layer created initially as a by-product could have been co-opted for competence.

## Future Directions

The investigations described here and the subsequent advances in knowledge have laid a firm foundation for determining the molecular mechanisms for DNA uptake by all type IV filaments. In particular, the recent advances in cryo-electron tomography show great promise for determining the structures of the large multi-protein complexes of the competence machinery in their native context. Identification of the T4F DNA receptors, the equivalents of ComP in *Neisseria*, is another crucial step for distinguishing between the mechanistic possibilities described here; particularly in terms of how DNA is localized on the pilus fiber. The complexities of how pilin subunits are organized within pili will have a significant impact on these studies in cases where the DNA receptor is a pilin like ComP; investigations into *P. aeruginosa* T4P have shown that minor pilin subunits may form a tip complex and yet also be incorporated sporadically throughout the filament (Giltner et al., 2010; Nguyen et al., 2015).

The knowledge that three distinct but related types of filaments all mediate natural competence also provides encouragement that knowledge gained in one system may help to drive progress in the others. However the similarities between T4F do not necessarily imply a common mechanism of action; the ability of T4P to promote DNA-binding in species which are not naturally competent (van Schaik et al., 2005), which is likely to play a role in biofilm formation (Petersen et al., 2005), suggests that the evolution of type IV pili, including DNA binding, may have been driven by adhesion rather than natural competence. Subsequently, natural competence may have developed in various T4F-producing bacteria through distinct molecular mechanisms. In conclusion, the study of DNA-uptake by type IV filaments promises to expand our understanding, not only of bacterial natural competence, but the evolutionary mechanisms by which competence arose.

## Author Contributions

KP conceived and wrote the manuscript, made the 3D models and all figures.

### Conflict of Interest Statement

The author declares that the research was conducted in the absence of any commercial or financial relationships that could be construed as a potential conflict of interest.
